# The Role of Sleep Studies to Predict Post-Operative Outcomes in Children with Medical Complexity

**DOI:** 10.3390/children13091170

**Published:** 2026-08-30

**Authors:** Simona Basilicata, Marialaura Marrazzo, Francesca Peri, Sergio Ghirardo, Massimo Maschio, Melissa Borrelli, Raffaella Sagredini, Gaia Milvia Bregant, Francesca Vittoria, Marco Carbone, Alessandro Amaddeo

**Affiliations:** 1 Department of Translational Medical Sciences, Pediatric Pulmonology, Federico II University, 80131 Naples, Italy; 2 Department of Medicine, Surgery and Health Sciences, University of Trieste, 34127 Trieste, Italy; 3 IRCCS Burlo Garofolo of Trieste, Institute for Maternal and Child Health, 34100 Trieste, Italy

**Keywords:** children with medical complexity, sleep-disordered breathing, postoperative respiratory complications

## Abstract

**Background:** In children with medical complexity (CMC), respiratory involvement represents one of the main causes of morbidity. Early recognition of nocturnal hypoventilation or sleep-disordered breathing (SDB) may allow timely intervention and reduce perioperative respiratory complications. This study aimed to evaluate the role of sleep studies in guiding clinical decision-making and perioperative respiratory management in CMC patients undergoing spinal arthrodesis for scoliosis correction. **Methods:** We retrospectively analysed clinical data from 357 patients (196 females) who underwent scoliosis surgery between 20 November 2012 and 11 June 2024. All patients received a standardized preoperative respiratory assessment at the Pulmonology Unit of IRCCS Burlo Garofolo. **Results**: Patients were classified into seven diagnostic groups, including neurological, neuromuscular, cerebral palsy, malformative, and other complex conditions. Median age at surgery was 12 years (range 2–22). Sleep studies were performed in 119 patients and identified abnormalities suggestive of sleep-disordered breathing or nocturnal hypoventilation, including elevated apnea–hypopnea index, oxygen desaturation, or abnormal transcutaneous carbon dioxide levels. Before surgery, 20 patients were receiving non-invasive ventilation *n*IV), initiated based on sleep study findings or pulmonary function impairment, while two patients were supported with continuous positive airway pressure (CPAP). Median Paediatric Intensive Care Unit (PICU) stay was 4.0 days (IQR 3.0–5.5 days; range 1–23), with no statistically significant differences across diagnostic categories (*p* = 0.12). Postoperatively, respiratory support was frequently required, including invasive mechanical ventilation, NIV, or CPAP, but the duration of invasive ventilation was 15.5 h (range 1–36). Only one patient required tracheostomy, and no perioperative deaths occurred. **Conclusions:** In CMC undergoing spinal arthrodesis, preoperative sleep studies significantly influence the clinical decision-making process and support perioperative respiratory assessment by identifying occult respiratory vulnerability and informing tailored ventilatory strategies. While associated with structured management pathways, the prospective impact of sleep-guided interventions on postoperative clinical outcomes remains to be confirmed through controlled studies.

## 1. Introduction

Children with Medical Complexity (CMC) represent a heterogeneous group of patients affected by congenital or acquired chronic conditions characterized by multisystem involvement, medical fragility, and high healthcare needs. These children often require coordinated, multidisciplinary care due to the coexistence of neurological, respiratory, gastrointestinal, orthopaedic, and cardiovascular comorbidities, with a significant impact on morbidity, healthcare utilization, and quality of life [1].

Among the multiple organ systems involved, respiratory impairment represents one of the leading causes of morbidity and hospitalization in CMC. Chronic respiratory insufficiency frequently develops as a result of neuromuscular weakness, impaired airway clearance, altered chest wall mechanics, and recurrent respiratory infections. Early identification of nocturnal hypoventilation or sleep-disordered breathing (SDB) is crucial, as these conditions often precede the onset of clinically evident daytime respiratory failure [2,3]. Timely recognition allows the initiation of proactive respiratory interventions, such as non-invasive ventilation *n*IV), potentially preventing respiratory exacerbations and reducing long-term respiratory morbidity [4].

Scoliosis is a highly prevalent comorbidity in CMC, particularly among those with neurological disorders, neuromuscular diseases, cerebral palsy, and malformative syndromes. The prevalence of scoliosis varies according to the underlying condition but may reach up to 90% in children with severe cerebral palsy [5] and increases progressively with age in disorders such as Duchenne muscular dystrophy [6,7,8]. In this vulnerable population, scoliosis represents not only a musculoskeletal deformity but also a major contributor to respiratory dysfunction. Progressive spinal curvature may lead to thoracic insufficiency syndrome, characterized by reduced thoracic volume, impaired chest wall compliance, and restricted lung growth, ultimately resulting in restrictive lung disease and worsening gas exchange [9].

Management of scoliosis in CMC follows a progressive approach. Conservative strategies, including bracing, are commonly employed in early stages to slow curve progression. However, bracing does not reverse established deformities and may become ineffective as neuromuscular weakness progresses. Moreover, prolonged bracing can negatively impact comfort, mobility and, in some cases, respiratory mechanics by limiting chest wall expansion. For patients with severe or rapidly progressive scoliosis, vertebral arthrodesis remains the definitive surgical treatment. When successful, spinal fusion aims to stabilize the deformity, improve postural alignment, and potentially prevent further deterioration of respiratory function.

Despite its benefits, spinal arthrodesis in CMC is a major surgical procedure associated with a substantial risk of perioperative complications, with respiratory complications being the most frequent and clinically relevant. Postoperative respiratory morbidity may include prolonged mechanical ventilation, need for non-invasive ventilatory support, respiratory infections, and extended paediatric intensive care unit (PICU) stays [10,11]. These risks are particularly pronounced in patients with pre-existing respiratory vulnerability, highlighting the importance of careful preoperative assessment and optimization.

Identifying patients at higher risk of developing postoperative respiratory complications would allow the implementation of targeted proactive strategies, such as early initiation of ventilatory support, respiratory physiotherapy, and tailored perioperative monitoring. Such approaches have been shown to improve outcomes, particularly in neuromuscular populations, by stabilizing respiratory function and reducing postoperative morbidity [12]. While pulmonary function tests (PFTs) such as spirometry represent a standard component of routine preoperative respiratory evaluation, their clinical utility in CMC is frequently limited by severe cognitive, neurological, or motor impairments that prevent patients from performing required forced expiratory maneuvers. Consequently, passive and objective diagnostic tools—specifically nocturnal polygraphy and transcutaneous capnography—are increasingly necessary to accurately identify occult nocturnal hypoventilation and SDB in non-cooperative pediatric populations.

Assessment of nocturnal hypoventilation and SDB represents a valuable opportunity to detect early respiratory impairment before the onset of daytime symptoms. Sleep studies, including respiratory polygraphy and nocturnal capnography, allow the identification of alveolar hypoventilation, oxygen desaturation, and obstructive or central respiratory events, which may serve as surrogate markers of impending respiratory failure [13]. In neuromuscular diseases, sleep-related respiratory abnormalities often precede abnormalities on pulmonary function tests, supporting their role in early risk stratification and clinical decision-making.

However, despite the recognized burden of respiratory morbidity in CMC undergoing scoliosis surgery, evidence supporting structured preoperative respiratory risk stratification remains limited [9]. In particular, the role of sleep studies as part of routine preoperative assessment has not been clearly defined in this population. The existing literature has largely focused on neuromuscular cohorts or on overall postoperative complication rates [10,11], often without addressing how preoperative sleep-related respiratory abnormalities influence perioperative respiratory management and postoperative outcomes, despite evidence supporting their role as early markers of respiratory insufficiency in non-surgical settings [13,14]. Moreover, data derived from large, heterogeneous real-world cohorts of CMC remain scarce [1]. As a result, the clinical value of sleep studies in guiding respiratory decision-making before spinal arthrodesis, as well as their potential role in anticipating postoperative ventilatory needs, remains insufficiently characterized, particularly outside disease-specific neuromuscular cohorts and large administrative datasets [13,14,15].

To address these knowledge gaps, the primary objective of the present study was to evaluate the clinical utility of preoperative respiratory workups—specifically sleep studies—in predicting perioperative respiratory management and postoperative outcomes in a large single-center cohort of CMC undergoing spinal fusion for scoliosis correction. We hypothesized that preoperative sleep studies would identify previously underdiagnosed nocturnal hypoventilation and SDB, thereby directly guiding targeted perioperative ventilatory support and mitigating postoperative pulmonary morbidity.

## 2. Materials and Methods

### 2.1. Study Design and Population

We conducted a retrospective observational study using routinely collected clinical data from paediatric patients evaluated at the Institute for Maternal and Child Health—IRCCS Burlo Garofolo, Trieste, Italy. CMC were defined according to the framework proposed by Cohen et al. [1] as patients affected by chronic conditions involving multiple organ systems, associated with medical fragility and increased healthcare needs.

All CMC patients who underwent spinal arthrodesis for scoliosis correction between 20 November 2012, and 11 June 2024, were eligible for inclusion. All patients underwent a standardized preoperative respiratory assessment performed by the Pulmonology Unit as part of the multidisciplinary surgical pathway.

Exclusion criteria were defined prior to study enrollment. Patients were excluded if they met any of the following conditions: severe anesthesia-related or cardiovascular risk requiring specialized facilities not available at our center (e.g., pediatric cardiac surgery or neurosurgery), extreme orthopedic complexity, or high predicted respiratory risk leading to post-operative tracheostomy dependence.

### 2.2. Data Collection

For each patient, demographic and clinical data were collected, including date of birth, sex, date of surgery, age at surgery, body mass index (BMI) prior to surgery, and surgical approach. Clinical comorbidities and respiratory risk factors were recorded, including gastrostomy tube (G-tube) dependency, history of Nissen fundoplication, previous sputum cultures, frequency of respiratory infections in the year preceding surgery (defined as the need for antibiotic therapy), and baseline use of non-invasive ventilation *n*IV) or continuous positive airway pressure (CPAP), including the clinical indications for its initiation. Preoperative pulmonary function tests were recorded when feasible, specifically forced expiratory volume in 1 s (FEV_1_) and forced vital capacity (FVC), expressed as percentage of predicted values.

### 2.3. Preoperative Sleep Testing and Respiratory Assessment

Preoperative sleep studies were targeted based on specific clinical indications, including reported symptoms suggestive of SDB or nocturnal hypoventilation, severe baseline restrictive ventilatory impairment on spirometry (FVC < 50% predicted), upper airway anatomical abnormalities, or underlying etiologies carrying a high risk for nocturnal respiratory compromise (e.g., neuromuscular disorders or severe cerebral palsy).

Sleep testing comprised nocturnal cardiorespiratory polygraphy combined with continuous transcutaneous capnography (SenTec SDMS, SenTec AG, Therwil, Switzerland), tailored to patient characteristics and feasibility. All recordings were scored manually following standard criteria in accordance with the American Academy of Sleep Medicine (AASM) guidelines [16]. Collected sleep parameters included the apnea–hypopnea index (AHI), oxygen desaturation index (ODI), mean and minimum oxygen saturation (SpO_2_), percentage of sleep time spent with SpO_2_ < 90%, mean and peak transcutaneous carbon dioxide (tcPCO_2_) values, and percentage of sleep time spent with tcPCO_2_ > 50 mmHg. SDB was classified based on AHI thresholds, with obstructive sleep apnea (OSA) defined as mild (AHI > 1 to 5 events/hour), moderate (AHI > 5 to 10 events/hour), and severe (AHI ≥ 10 events/hour) [17]. Nocturnal hypoventilation was defined as tcPCO_2_ > 50 mmHg for >2% of total sleep time, or a peak tcPCO_2_ > 50 mmHg, according to standard ERS technical criteria [17]. Significant nocturnal desaturation was defined as SpO_2_ < 90% or SpO_2_ < 88% for ≥5 consecutive minutes [16].

### 2.4. Study Outcomes

The primary outcomes of this study were the length of stay in the PICU and the overall duration of postoperative mechanical ventilation. Secondary outcomes included the need for postoperative non-invasive respiratory support *n*IV or CPAP) and the requirement for respiratory rehabilitation strategies (e.g., cough assist devices or intrapulmonary percussive ventilation, IPV).

### 2.5. Statistical Analysis

Continuous variables were tested for normality of distribution using the Shapiro–Wilk test. Normally distributed continuous data are reported as mean ± standard deviation (SD), whereas non-normally distributed continuous variables—including PICU length of stay (LOS) and mechanical ventilation duration—are presented as median and interquartile range (IQR, 25th–75th percentiles) alongside the overall range (minimum–maximum). Categorical variables are presented as absolute numbers and percentages *n*, %). Comparisons of continuous variables between groups were evaluated using the Mann–Whitney U test or Kruskal–Wallis H test across diagnostic categories, as appropriate. Categorical variables were compared using the Chi-square test or Fisher’s exact test. Multivariable regression analyses were not performed due to subgroup sample size constraints and the exploratory retrospective design of the study. A two-tailed *p*-value < 0.05 was considered statistically significant for all analyses. Statistical analyses were performed using Python software (version 3.10, Python Software Foundation) with SciPy statistical packages.

### 2.6. Ethical Considerations

Ethical Committee approval was not required, as the General Authorization to Process Personal Data for Scientific Research Purposes (Authorization no. 9/2014) states that retrospective archive studies using ID codes, which prevent data from being traced back directly to the subject, do not need ethics approval (18). Parents provided informed consent at the first visit, agreeing that “clinical data may be used for clinical research purposes, epidemiology, the study of pathologies, and training, to improve knowledge, care, and prevention.”

The Institutional Review Board (IRB) approved the study (RC10/21).

## 3. Results

### 3.1. Demographic and Clinical Characteristics

A total of 371 consecutive pediatric patients evaluated for surgical treatment of complex scoliosis were considered for study inclusion. Of these, 14 patients were deemed unsuitable for spinal arthrodesis during the preoperative assessment and were excluded prior to surgery. The excluded patients belonged to the following primary diagnostic categories: neurological disorders *n* = 6), other underlying conditions *n* = 4), cerebral palsy *n* = 2), neuromuscular diseases *n* = 1), and neurofibromatosis *n* = 1). Following multidisciplinary evaluation, the specific clinical reasons for exclusion, stratified by baseline etiology, were as follows:

- Fragile general conditions/high anesthetic risk *n* = 4): 3 patients with neurological disorders and 1 with cerebral palsy;
- Cardiac or thoracic comorbidities *n* = 4): 3 patients categorized under other conditions and 1 with neurofibromatosis, presenting with potential perioperative complications that could not be managed at our institution due to the absence of an on-site cardiothoracic surgery department;
- Excessive scoliosis severity *n* = 4): 2 patients with neurological disorders, 1 with cerebral palsy, and 1 with another condition, in whom deformity severity exceeded safe surgical correction limits;
- Requirement for specialized neurosurgical backup *n* = 1): 1 patient with a neurological disorder presenting with severe hydrocephalus excluded due to the absence of an on-site neurosurgery department;
- Severe baseline respiratory impairment *n* = 1): 1 patient with neuromuscular disease presenting with pulmonary dysfunction judged incompatible with surgery.

**Figure 1:**
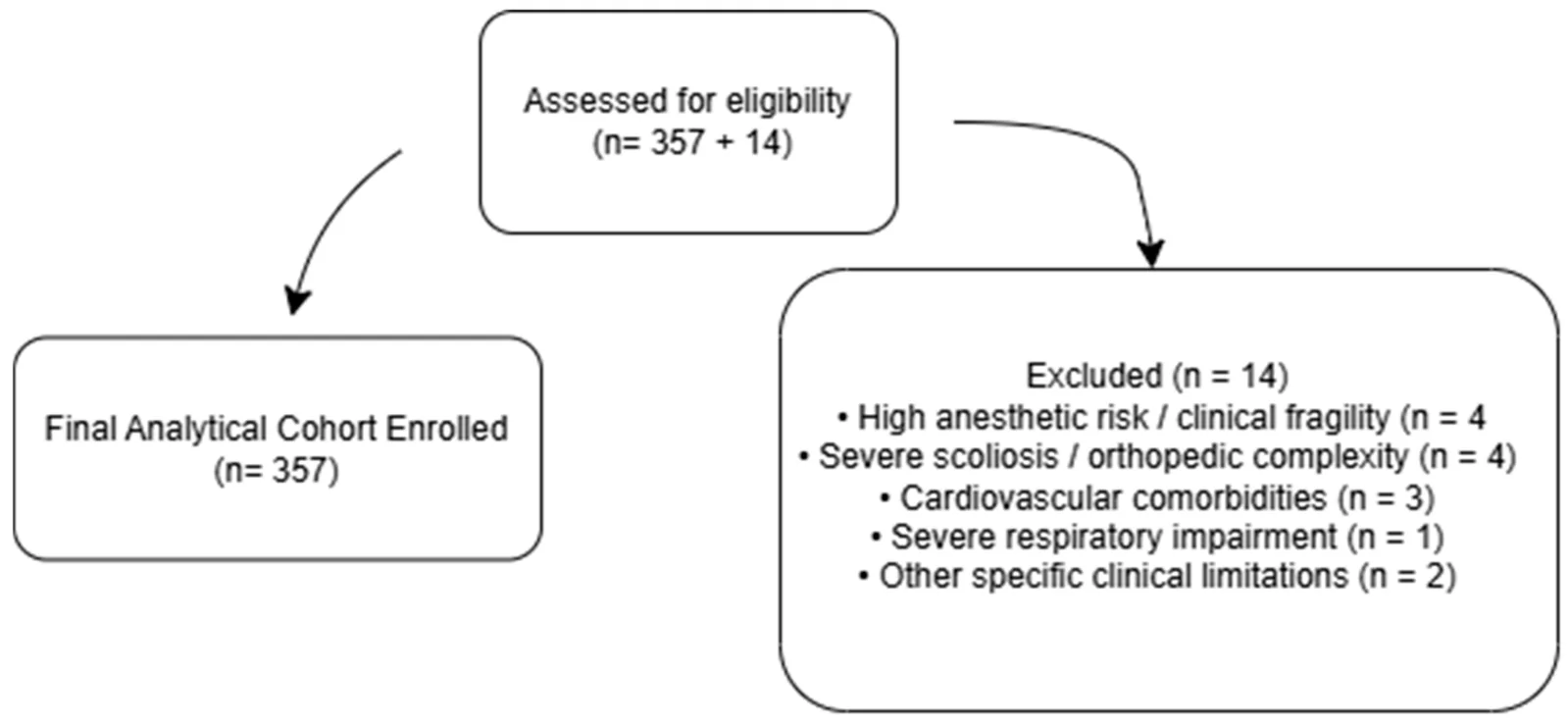
Patient selection flow diagram. Flowchart illustrating patient evaluation, specific reasons for preoperative exclusion, and final cohort enrollment.

The patient selection flow, reasons for exclusion, and final cohort distribution are summarized in Figure 1 and Table 1.

The final analytical cohort comprised 357 patients who successfully underwent spinal arthrodesis. The cohort included 196 females (55%) and 161 males (45%). Median age at surgery was 12 years (range 2–22), and mean body mass index (BMI) was 17.49 kg/m^2^ (range 10.18–30.6). Patients were classified according to the underlying condition into seven groups: neurological disorders *n* = 101, 28%), neuromuscular diseases *n* = 34, 10%), cerebral palsy *n* = 36, 10%), malformative syndromes *n* = 57, 16%), neurofibromatosis *n* = 24, 7%), Rett syndrome *n* = 10, 3%), and other conditions *n* = 95, 27%) (Table 2).

**Figure 2:**
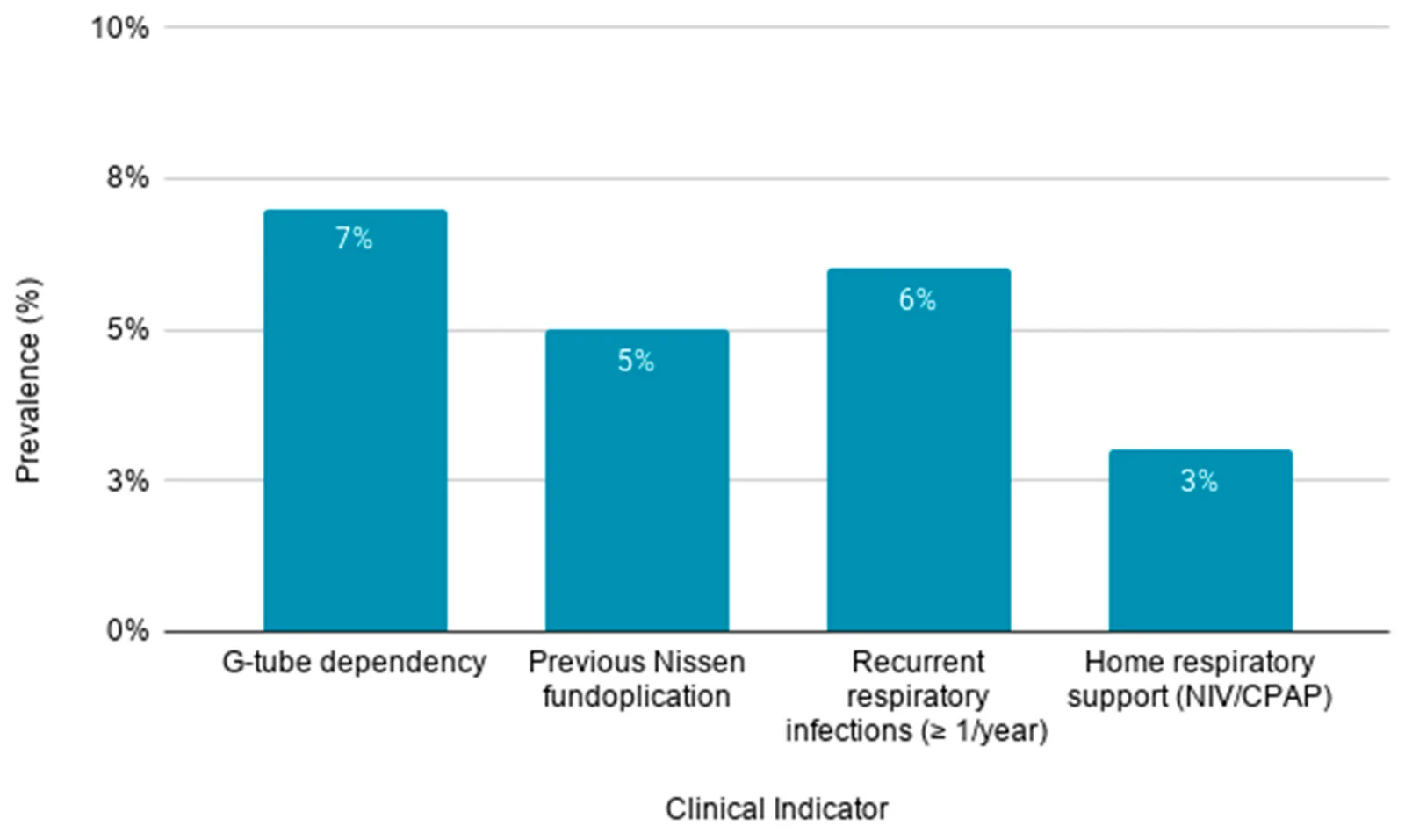
Prevalence of baseline clinical indicators and respiratory comorbidities in the study population *n* = 357). Abbreviations: G-tube, gastric tube; NIV, non-invasive ventilation; CPAP, continuous positive airway pressure.

The prevalence of key baseline clinical indicators and comorbidities—specifically gastrostomy tube (G-tube) dependency (24 patients, 7%), previous Nissen fundoplication (18 patients, 5%), recurrent respiratory infections (21 patients, 6%), and home ventilatory support (10 patients, 3%)—is visually summarized in Figure 2.

### 3.2. Preoperative Respiratory Assessment and Ventilation Management

**Figure 3:**
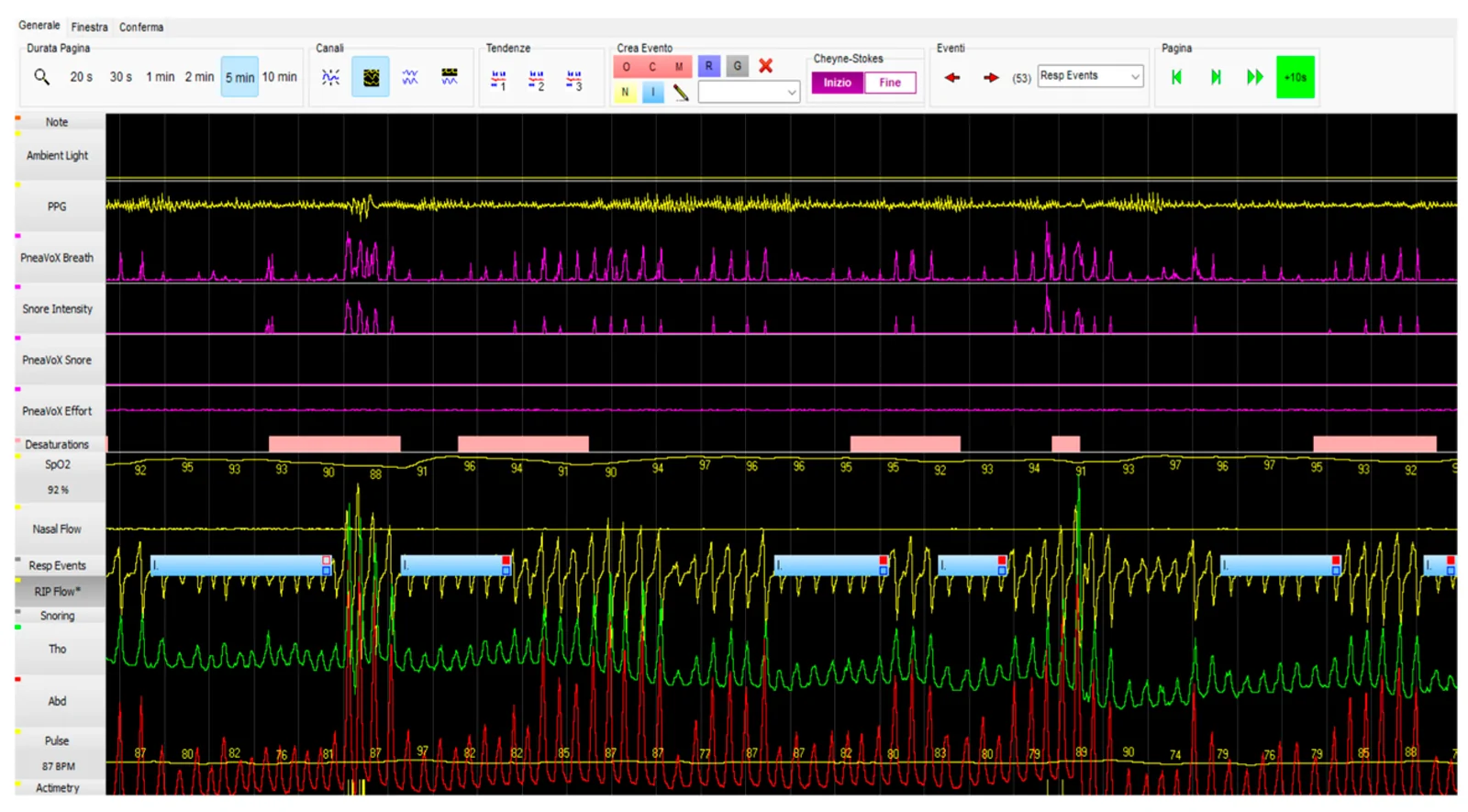
Preoperative nocturnal polygraphy tracing of a pediatric patient with a neuromuscular disorder exhibiting SDB. The panels from top to bottom display: photoplethysmography (PPG) waveform; PneaVoX respiratory breath, snore intensity, snore, and effort channels; oxygen desaturation event detection (highlighted by pink blocks); hemoglobin oxygen saturation (SpO_2_) trend line; nasal airflow; scored respiratory events (marked as ‘I’ for hypopneas); respiratory inductance plethysmography (RIP) flow*; thoracic (Tho, green) and abdominal (Abd, red) respiratory effort; and pulse rate (BPM). The tracing illustrates cyclic reductions in respiratory airflow and effort accompanied by repetitive oxygen desaturations, secondary to progressive respiratory muscle weakness. *RIP flow indicates the airflow signal derived from thoraco-abdominal respiratory inductance plethysmography bands)

**Figure 4:**
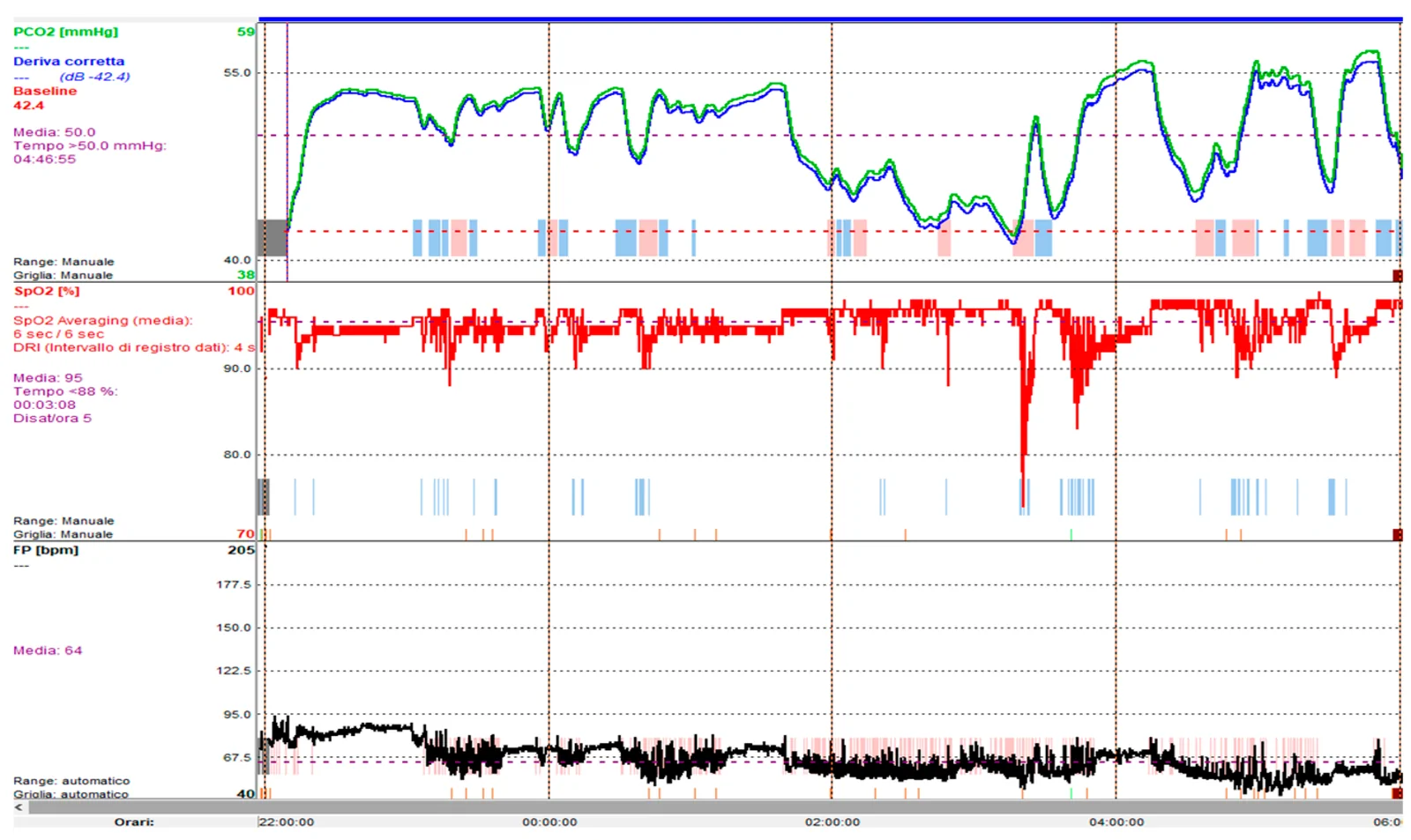
Preoperative nocturnal transcutaneous capnography and oximetry monitoring profile demonstrating nocturnal hypoventilation. The top panel shows the transcutaneous carbon dioxide (tcPCO_2_, green/blue lines) trend, highlighting sustained nocturnal hypercapnia with a mean value of 50.0 mmHg and prolonged time spent above 50 mmHg (4 h and 46 min). The middle panel illustrates the hemoglobin oxygen saturation (SpO_2_, red line) trend with visible clusters of acute desaturations. The bottom panel displays the continuous pulse rate (FP, black line) trend.

All patients underwent preoperative pneumological evaluation. No patient had diurnal hypercapnia at capillary blood gases analysis. Pulmonary function tests were feasible in 57 patients. Mean forced expiratory volume in 1 s (FEV_1_) was 71.5% of predicted (range 36–120%), and mean forced vital capacity (FVC) was 72.52% of predicted (range 35–120%). Sleep studies were performed in 119 patients (Figure 3 and Figure 4). Among these, 11 patients (9%) had an apnea–hypopnea index (AHI) > 5 events/hour, 10 (8%) had an oxygen desaturation index (ODI) > 10 events/hour, 19 (16%) had a minimum oxygen saturation (SpO_2_) < 88%, and 4 patients (3%) spent more than 2% of total sleep time with SpO_2_ < 90%. Four patients showed transcutaneous carbon dioxide (tcPCO_2_) values > 50 mmHg for more than 2% of total sleep time (Table 3).

Before surgery, 20 patients were receiving NIV. In eight of these patients, NIV was initiated based on sleep study findings, while in one case NIV was started due to reduced pulmonary function test values. Two patients were supported with CPAP, in one case initiated following pneumological evaluation.

### 3.3. Surgical Management and Postoperative Respiratory Outcomes

Regarding surgical approach, posterior spinal fusion was performed in 350 patients, anterior fusion in three patients, and a combined anterior–posterior approach in four patients. Data on PICU length of stay were available for 232 patients with complete anesthesiological discharge documentation. For the overall analyzed cohort, the median PICU stay was 4.0 days (IQR: 3.0–5.5 days; range: 1–23 days). When stratified by underlying pathology, median PICU LOS was 5.0 days in patients with cerebral palsy (IQR: 4.0–6.0), 5.0 days in neuromuscular diseases (IQR: 3.5–6.0), 5.0 days in malformative syndromes (IQR: 3.5–6.0), 6.0 days in Rett syndrome (IQR: 5.0–6.8), 4.0 days in neurological disorders (IQR: 3.0–6.0), 4.0 days in neurofibromatosis (IQR: 3.0–5.0), and 4.0 days in other conditions (IQR: 3.0–5.0). No statistically significant difference in PICU LOS was observed across diagnostic categories (*p* = 0.12).

Thirty-three patients required invasive mechanical ventilation, 48 required NIV, and 22 were supported with CPAP. Only one patient required tracheostomy after three failed extubation attempts. Mean duration of invasive ventilation was 15.56 h (range 1–36). As part of postoperative respiratory rehabilitation in the PICU, 29 patients were treated with cough assist devices and nine with IPV (Table 4).

To further explore differences in postoperative respiratory outcomes according to underlying conditions, a subgroup analysis was performed across diagnostic categories.

Patients with neurological disorders *n* = 101) showed a median PICU length of stay of 4.0 days (IQR: 3.0–6.0). Thirteen patients (13%) required invasive mechanical ventilation, 12 (12%) required NIV and 5 (5%) were supported with CPAP. In the neuromuscular disease *n*MD) group *n* = 34), median PICU length of stay was 5.0 days (IQR: 3.5–6.0). Five patients (15%) required invasive mechanical ventilation, 12 (35%) required NIV, and no patients required CPAP. One patient was chronically ventilator-dependent via tracheostomy placed during infancy.

Patients with cerebral palsy *n* = 36) had a median PICU length of stay of 5.0 days (IQR: 4.0–6.0). Five patients (14%) required invasive ventilation and six (17%) required NIV, while no patients required CPAP. In patients with malformative syndromes *n* = 57), median PICU length of stay was 5.0 days (IQR: 3.5–6.0). Three patients (5%) required invasive ventilation, four (7%) required NIV, and five (9%) required CPAP. Among patients with neurofibromatosis *n* = 24), median PICU length of stay was 4.0 days (IQR: 3.0–5.0). No patients required invasive ventilation, while two (8%) required NIV and three (13%) required CPAP. Patients with Rett syndrome *n* = 10) showed a longer median PICU length of stay of 6.0 days (IQR: 5.0–6.8). Three patients (30%) required invasive ventilation, two (20%) required NIV, and three (30%) required CPAP. One patient required tracheostomy. In the group of patients with other conditions *n* = 95), median PICU length of stay was 4.0 days (IQR: 3.0–5.0). Four patients (4%) required invasive ventilation, nine (9%) required NIV, and six (6%) required CPAP. One patient was chronically ventilator-dependent via tracheostomy placed at birth. No perioperative deaths were observed in all groups.

To directly address the prognostic impact of preoperative sleep study findings, a comparative outcome analysis was performed in the sleep study cohort *n* = 119), comparing patients with SDB/hypoventilation (SDB group, *n* = 10) to those with normal sleep parameters *n* = 109). Overall, postoperative clinical outcomes were comparable between the two groups. Median PICU length of stay was 5.0 days (IQR: 4.0–6.0) in the SDB group versus 6.0 days (IQR: 4.0–7.0) in the normal sleep study group (*p* = 0.24). Postoperative invasive mechanical ventilation was required in 10.0% (1/10; duration: 36 h) of SDB patients compared to 13.8% (15/109; median duration: 12 h, range: 6–24) of normal sleep patients (*p* = 1.00). Notably, 100% (10/10) of patients in the SDB group successfully received postoperative non-invasive ventilatory support *n*IV/CPAP)—which had been initiated or optimized preoperatively based on pneumological screening—compared to 75.2% (82/109) in the normal group (*p* = 0.081). Regarding severe respiratory complications, zero (0/10, 0%) unplanned re-intubations or tracheostomies occurred in the SDB group. Conversely, in the normal sleep study group, one patient (1/109, 0.9%) experienced post-extubation failure requiring unplanned re-intubation on postoperative day 4 and subsequent tracheostomy following three failed extubation attempts (*p* = 1.00).

Additionally, the specific AASM-scored sleep parameters detailed in the Methods—including minimum SpO_2_, nocturnal hypoventilation (tcPCO2 >50 mmHg for >2% of sleep time), AHI, and ODI—were explicitly evaluated against postoperative outcomes. None of these nocturnal metrics demonstrated a statistically significant association with prolonged mechanical ventilation, increased PICU length of stay, or post-extubation respiratory failure (all *p* > 0.05).

## 4. Discussion

The present study provides evidence on the role of preoperative sleep studies in the respiratory management of CMC undergoing spinal arthrodesis for scoliosis correction. In a large, heterogeneous cohort, our findings show that structured preoperative respiratory assessment, including systematic sleep studies, allows early identification of patients with incipient respiratory impairment and contributes to guiding perioperative ventilatory strategies. Despite the high prevalence of underlying respiratory vulnerability, postoperative respiratory outcomes were favorable, with low rates of prolonged invasive ventilation, rare need for tracheostomy, and absence of perioperative mortality.

Importantly, sleep studies influenced clinical decision-making beyond traditional pulmonary function testing. In our cohort, sleep-related respiratory abnormalities supported the initiation of NIV or CPAP before surgery in selected patients, even in the absence of overt daytime respiratory failure. This proactive approach guided respiratory optimization during the perioperative period, illustrating how nocturnal hypoventilation and SDB represent clinically meaningful markers to inform perioperative planning and risk stratification in children with medical complexity undergoing major spinal surgery.

Our postoperative outcomes are consistent with previously published data on paediatric spinal deformity surgery, reporting comparable PICU length of stay and low mortality rates, even in high-risk populations [18,19]. Studies focusing on neuromuscular and congenital scoliosis have highlighted respiratory complications as a major determinant of postoperative morbidity and resource utilization [10,11,20]. In line with this literature, a substantial proportion of patients in our cohort required postoperative respiratory support. However, the relatively short duration of invasive ventilation and the low incidence of severe complications suggest that careful preoperative evaluation is associated with structured multidisciplinary management, even in medically fragile patients.

In addition, the subgroup analysis across diagnostic categories provided further insight into the heterogeneity of postoperative respiratory outcomes. While overall outcomes were broadly comparable, some clinically relevant differences emerged. Patients with neuromuscular diseases and Rett syndrome tended to require higher levels of ventilatory support, particularly in terms of NIV and invasive mechanical ventilation, whereas patients with malformative syndromes and neurofibromatosis showed lower rates of invasive respiratory support. These differences likely reflect the underlying pathophysiological characteristics of each condition, with neuromuscular weakness predisposing to impaired ventilatory reserve and a higher susceptibility to postoperative respiratory decompensation. Conversely, patients without primary respiratory muscle involvement may better tolerate perioperative stress despite the presence of complex creomorbidities.

Notably, despite these variations in respiratory support requirements, no consistent differences were observed in severe outcomes such as tracheostomy or mortality across diagnostic groups, supporting the overall safety of the multidisciplinary perioperative approach adopted in this cohort.

The integration of sleep studies into the preoperative assessment pathway has relevant clinical implications. By identifying early signs of nocturnal hypoventilation or SDB, clinicians can anticipate respiratory needs and implement targeted interventions before surgery, rather than reacting to postoperative deterioration. This proactive strategy aligns with recent evidence emphasizing the key role of sleep monitoring in identifying subclinical hypoventilation and timing respiratory support in pediatric complex care [21], supporting a paradigm of perioperative respiratory ‘prehabilitation’ where preoperative NIV optimization stabilizes chest wall mechanics, recruits atelectatic lung areas, and improves fatigue tolerance prior to surgical stress [3,22]. In our experience, this approach facilitated individualized perioperative planning, including allocation of PICU resources and early initiation of postoperative respiratory support when needed.

In our study, 11 patients were started on NIV/CPAP preoperatively and sleep studies represented the indication to treatment in 10 out of 11. Even if the proportion of patients with moderate to severe SDB was relatively low, sleep studies successfully identified those patients with sleep abnormalities who benefitted from timely respiratory intervention.

This proactive strategy likely contributed to favorable clinical outcomes. When comparing patients with preoperative sleep abnormalities (SDB group, *n* = 10) to those with normal sleep studies *n* = 109), no significant differences were observed in PICU length of stay or duration of invasive ventilation. Crucially, despite their baseline respiratory control vulnerability, patients in the SDB group experienced zero (0%) unplanned re-intubations or tracheostomies. In contrast, the single severe respiratory failure in our cohort—requiring re-intubation on postoperative day 4 and ultimately tracheostomy after three failed extubation attempts—occurred in the normal sleep study group. The observation that all patients in the SDB group were effectively managed with targeted postoperative NIV/CPAP (initiated or tailored preoperatively) points toward a potential benefit of systematic pneumological screening in mitigating baseline respiratory risk, potentially helping compromised children achieve a safety profile comparable to non-compromised peers.

Respiratory physiotherapy was documented in a subset of patients; however, given the retrospective nature of the study, its use may have been underreported in clinical records, particularly in patients routinely managed with airway clearance strategies.

In our cohort, 119 out of 357 patients (33.3%) underwent preoperative sleep studies. Rather than a universal screening strategy, this selective approach reflects pragmatic, real-world clinical decision-making. Sleep studies were performed based on specific clinical indications; consequently, the 119 patients who received sleep studies represented a clinically higher-risk subgroup. These individuals were preferentially selected due to pre-existing conditions predisposing to nocturnal hypoventilation or SDB—such as neuromuscular diseases, cerebral palsy, Rett syndrome, severe chest wall deformities, or anatomical upper airway abnormalities predisposing to SDB—or because of reported symptoms suggestive of nocturnal respiratory impairment. Conversely, patients who did not undergo sleep studies (two-thirds of the cohort) were primarily individuals with idiopathic scoliosis, preserved baseline neurological status, and low clinical suspicion of nocturnal hypoventilation, for whom routine sleep studies were not deemed clinically necessary.

Importantly, abnormal sleep study parameters scored according to pediatric AASM criteria, including minimum SpO_2_, AHI, ODI, and nocturnal hypoventilation, were not significantly associated with worse postoperative outcomes, such as prolonged mechanical ventilation or extended PICU length of stay. While this lack of statistical association could theoretically suggest a limited predictive yield of sleep studies, an alternative and clinically plausible explanation is the potential risk-mitigating effect of proactive perioperative management. In our clinical pathway, preoperative sleep studies enabled early identification of vulnerable patients, guiding the timely initiation or optimization of non-invasive respiratory support *n*IV/CPAP) prior to surgery. Consequently, pre-emptively addressing nocturnal hypoventilation may have helped attenuate the baseline excess respiratory risk in these fragile individuals, potentially contributing to postoperative outcomes comparable to those of lower-risk peers. Nevertheless, given the retrospective nature of our cohort, this risk-mitigation hypothesis remains observational and warrants further confirmation in prospective, multi-center studies.

These findings support a multidisciplinary perioperative pathway involving pulmonologists, orthopedic surgeons, anesthesiologists and intensivists. Sleep studies may complement pulmonary function testing, particularly in children unable to perform reliable spirometry or in those with neuromuscular weakness, where nocturnal hypoventilation often represents the earliest sign of respiratory muscle fatigue and precedes daytime respiratory failure, requiring targeted screening to guide timely intervention [13,21]. While universal sleep study screening may not be feasible or necessary for all patients, our data suggest that targeted use in clinically selected children with CMC can meaningfully inform risk stratification and perioperative respiratory management.

### Study Limitations

Several limitations of this study warrant consideration. Primarily, its retrospective, single-center design and relatively small sample size may limit the generalizability of our findings to centers with different resource levels or expertise. Furthermore, given the descriptive design, the absence of an untreated control cohort, and small diagnostic subgroup sizes that precluded multivariable regression analysis, our findings reflect unadjusted associations rather than direct causality; thus, residual confounding cannot be entirely excluded.

Concerning diagnostic methodologies, sleep testing was inherently heterogeneous: cardiorespiratory polygraphy combined with transcutaneous capnography was employed rather than full attended laboratory polysomnography (PSG). This choice was pragmatically guided by patient tolerance, age, and neurodevelopmental status in a population with high medical complexity. Likewise, formal spirometry could only be reliably performed in a minority of patients *n* = 57) due to severe underlying cognitive, motor, or neurological impairments, limiting systematic physiological correlation across the entire cohort.

With respect to database completeness, the exact interval between preoperative respiratory assessment and surgery was not systematically tracked; nevertheless, in routine clinical practice, these evaluations were consistently performed as part of the preoperative surgical workup, and approximately at least the 6 months before surgery in most of the cases. In addition, certain granular non-respiratory baseline parameters—such as individual Cobb angles, formal nutritional z-scores, baseline ambulatory status, and systematic cardiac metrics—were not uniformly recorded. Moreover, respiratory physiotherapy was documented in a subset of patients and may have been underreported due to the retrospective design.

Lastly, preoperative sleep studies were targeted based on clinical indications rather than implemented as universal screening across the entire cohort (119 out of 357 patients). Consequently, the analyzed group represents a selected, higher-risk subgroup, introducing an inherent selection bias that must be considered when interpreting our findings.

## 5. Conclusions

The present study highlights that a structured preoperative respiratory risk assessment, incorporating targeted sleep studies, plays a crucial role in perioperative management by directly influencing clinical decision-making in children with medical complexity undergoing spinal fusion. While baseline medical fragility in this population is high, early recognition of SDB and nocturnal hypoventilation enables timely ventilatory optimization, supporting perioperative respiratory pathways and contributing to favorable postoperative outcomes with reduced respiratory morbidity. Given the observational nature of our cohort, the direct impact of preoperative sleep studies on postoperative outcomes needs to be confirmed through future prospective, multi-center studies with standardized screening protocols to refine preoperative risk stratification criteria in this heterogeneous population.

## Figures and Tables

**Table 1:** Summary of patient selection, reasons for exclusion, and final cohort distribution.

Parameter/Category	No. of Patients (*n*)	Percentage (%)	Primary Etiology Breakdown
**Initially Screened Cohort**	**371**	**100.0%**	Pediatric patients evaluated for complex scoliosis
**Excluded Patients**	**14**	**3.8%**	*Detailed below by clinical reason*
**Final Study Cohort**	**357**	**96.2%**	Patients who underwent spinal arthrodesis
**Specific Clinical Reasons for Exclusion**			
**Fragile general conditions/High anesthetic risk**	4	28.6% *	Neurological disorders (*n* = 3), Cerebral palsy (*n* = 1)
**Cardiovascular or thoracic comorbidities**	4	28.6% *	Other underlying conditions (*n* = 3), Neurofibromatosis (*n* = 1)
**Excessive scoliosis severity**	4	28.6% *	Neurological disorders (*n* = 2), Cerebral palsy (*n* = 1), Other conditions (*n* = 1)
**Specialized neurosurgical backup required**	1	7% *	Neurological disorder with severe hydrocephalus (*n* = 1)
**Severe baseline respiratory impairment**	1	7% *	Neuromuscular disease (*n* = 1)

* Percentages for exclusion reasons are calculated out of the total excluded cohort (*n* = 14).

**Table 2:** Demographic and Clinical Characteristics of the Overall Population *n* = 357).

Characteristic/Variable	Age at Surgery, Years (Mean ± SD or Median [Range])	Patient Count, *n* (%)
**Demographics**		
Female sex	—	196 (55%)
Male sex	—	161 (45%)
Age at surgery, median (range)	12 (2–22)	357 (100%)
Body mass index (BMI, kg/m^2^), mean (range)	17.49 (10.18–30.6)	357 (100%)
**Diagnostic Categories**		
—*Neurological disorders*	15 ± 3.2	101 (28%)
—*Neuromuscular diseases*	14 ± 4.2	34 (10%)
—*Cerebral palsy*	16 ± 2.6	36 (10%)
—*Malformative syndromes*	12 ± 4.9	57 (16%)
—*Neurofibromatosis*	13 ± 3.7	24 (7%)
—*Rett syndrome*	14 ± 1.9	10 (3%)
—*Other conditions*	13 ± 5.0	95 (27%)
**Baseline Clinical Indicators & Comorbidities**		
Gastrostomy tube dependency	—	24 (7%)
Previous Nissen fundoplication	—	18 (5%)
Recurrent respiratory infections	—	21 (6%)
Home ventilatory support	—	10 (3%)

**Table 3:** Preoperative respiratory assessment.

Pulmonary Function Test, *n* = 57	Results
FEV_1_ (% predicted), mean (range)	71.5 (36–120)
FVC (% predicted), mean (range)	72.52 (35–120)
**Sleep studies performed, *n* =** 119	
AHI >5, *n* (%)	11 (9%)
ODI >10, *n* (%)	10 (8%)
Minimum SpO_2_ <88%, *n* (%)	19 (16%)
SpO_2_ <90% for >2% of TST, *n* (%)	4 (3%)
tcPCO_2_ >50 mmHg for >2% of TST, *n* (%)	4 (3%)

Abbreviations: FEV_1_, forced expiratory volume in 1 s; FVC, forced vital capacity; AHI, Apnea–hypopnea index; ODI, Oxygen desaturation index; SpO_2_, oxygen saturation; tcPCO_2_, transcutaneous carbon dioxide; TST, total sleep time.

**Table 4:** Postoperative respiratory and intensive care outcomes, and rehabilitation protocols *n* = 357).

Postoperative Outcome/Support	Value
**PICU Management**	
Length of stay (PICU LOS), (days), median [IQR]	4.0 (3.0–5.5)
**Respiratory Support**	
Invasive mechanical ventilation, *n*	33 (9%)
Duration of invasive ventilation, hours, mean (range)	15.56 (1–36)
NIV, *n* (%)	48 (13%)
CPAP, *n* (%)	22 (6%)
New tracheostomy requirement, *n* (%)	1 (<1%)
**Respiratory Rehabilitation**	
Mechanical cough assist device, *n* (%)	29 (8%)
IPV, *n* (%)	9 (3%)

Abbreviations: PICU, paediatric intensive care Unit; LOS, length of stay; IQR, interquartile range; NIV, non-invasive ventilation; CPAP, continuous positive airway pressure; IPV, Intrapulmonary percussive ventilation.

## Data Availability

The datasets generated and analyzed during the current study are not publicly available due to strict privacy and ethical restrictions regarding sensitive paediatric clinical data.

## Abbreviations

The following abbreviations are used in this manuscript:

AASM	American Academy of Sleep Medicine
AHI	Apnea-hypopnea index
BMI:	Body mass index
CMC	Children with medical complexity
CPAP	Continuous Positive Airway Pressure
FEV_1_	Forced expiratory volume in 1 s
FVC	Forced vital capacity
G-TUBE	Gastrostomy tube
IPV	Intrapulmonary percussive ventilation
IQR	Interquartile range
LOS	Length of stay
NIV	Non-invasive ventilation
NMD	Neuromuscular disease
ODI	Oxygen desaturation index
PICU	Paediatric intensive care unit
PT	Patient
SDB	Sleep-disordered breathing
SpO_2_	Haemoglobin oxygen saturation
SpO_2_ min	Minimum oxygen saturation
T90	Percentage of sleep time with SpO_2_ < 90%
tcPCO_2_	Transcutaneous carbon dioxide
TST	Total sleep time
